# Preclinical evaluation of MVA-MUC1-IL2 in combination with chemotherapy in murine models

**DOI:** 10.1186/2051-1426-1-S1-P90

**Published:** 2013-11-07

**Authors:** Micaël De Meyer, Isabelle Farine, Christelle Pichon, Ronald Rooke, Philippe Slos

**Affiliations:** 1 Oncology, Transgene SA, Illkirch-Graffenstaden, France

## 

The MUC1 glycoprotein is a major tumor associated antigen in human epithelial cancers in which it is overexpressed and hypoglycosylated, in contrast to the heavily glycosylated form of MUC1 found at lower expression level in normal cells. The TG4010 immunotherapeutic product consists of a suspension of attenuated vaccinia virus (Ankara strain), genetically modified to express MUC1 and the cytokine IL-2. The TG4010 immunotherapeutic agent is currently in advanced clinical development for non-small-cell lung cancer (NSCLC) in combination with chemotherapy. Antigen-specific models were generated by transfecting murine tumor cell lines with the human MUC1 and were used to address in vitro and in vivo issues regarding the combinatorial approach of chemotherapy and immunization with MVATG9931, an isogenic construct of TG4010. First, in vitro sensitivity to drugs used as standard of care to treat advanced NSCLC patients of the lymphoma RMA cell line and of the Lewis Lung Carcinoma cell line (LLC) expressing, or not, MUC1, was determined. The parental or transfected cell lines had similar sensitivity to gemcitabine, pemetrexed or the combination of these drugs with cisplatin. The in vivo anti-tumoral activity of chemotherapy was assessed in syngeneic C57BL/6 mice bearing tumors. In contrast to the in vitro situation, in vivo anti-tumoral activity of chemotherapy was clearly superior in the RMA or LLC MUC1-transfectants as compared to their respective un-transfected counterpart. Further investigations of the LLC model in vivo showed that MUC1 expression was linked to increased tumor aggressiveness when compared to the un-transfected LLC tumor with faster tumor growth and increased splenomegaly. Analysis of inflammatory cells and notably myeloid-derived suppressor cells (MDSC) indicated that their proportion was increased in the blood, spleen and tumor in mice bearing LLCMUC1 tumors as compared to that of mice with LLC tumors. Finally, using a gemcitabine-based regimen known to affect MDSC viability, combination therapy of LLCMUC1 tumors with MVATG9931 immunizations and gemcitabine/cisplatin increased significantly mice survival as compared to that of mice treated with chemotherapy alone. These preclinical data argues for a strong rationale to combine TG4010 with chemotherapy in advanced NSCLC patients.

